# A health economic analysis of osteoporotic fractures: who carries the burden?

**DOI:** 10.1007/s11657-013-0126-3

**Published:** 2013-02-19

**Authors:** Louise Hansen, Anne Sofie Mathiesen, Peter Vestergaard, Lars H. Ehlers, Karin D. Petersen

**Affiliations:** 1 Danish Center for Health Care Improvement, Aalborg University, Aalborg, Denmark; 2 Faculty of Medicine and Health Science Technologies, Aalborg University, Aalborg, Denmark; 3 Department of Endocrinology, Aalborg University, Aalborg, Denmark; 4 Fibigerstræde 4, 9220 Aalborg Ø, Denmark

**Keywords:** Costs and cost analysis, Osteoporosis, Fractures, Bone, Markov, Denmark

## Abstract

**Summary:**

This is a cost-of-illness study of osteoporotic fractures in Denmark estimating the incremental societal cost associated with osteoporotic fractures, with both direct cost and productivity cost. This study includes cost regarding hospitals, general practices, the patients, the municipalities and regions. The total cost of osteoporotic fractures in Denmark was estimated at EUR 1.563 billion.

**Purpose:**

The aim of this study is to estimate the societal burden imposed by osteoporotic fractures in Denmark. In contrast to prior studies, this study will present a comprehensive model for the cost of osteoporotic fractures regarding hospitals, general practices, the municipalities, the regions and the patients.

**Methods:**

This cost-of-illness study applied an incidence-based bottom-up approach from a societal perspective, including both direct costs and productivity costs. The study focused on incremental cost associated with osteoporotic fractures using a Markov model. Danish citizens ≥50 years with an osteoporotic fracture between 2001 and 2010 were studied.

**Results:**

The total cost of osteoporotic fractures in Denmark was estimated to EUR 1.563 billion in 2011, at EUR 628 million and EUR 936 million for men and women, respectively. The most expensive fracture for both genders was first hip fracture. The municipalities carried the majority of the costs, with 55–57 % of incremental lifetime cost.

**Conclusions:**

This study showed that the incremental societal burden of osteoporotic fractures is an important health problem. Medical costs of the osteoporotic fractures were substantial cost for the health care sector, but were by far exceeded by the cost for the municipality in terms of social services and rehabilitation.

**Electronic supplementary material:**

The online version of this article (doi:10.1007/s11657-013-0126-3) contains supplementary material, which is available to authorized users.

## Introduction

Osteoporosis is frequent [1], and its consequences, especially fractures [2–4] and disability [5], constitute a heavy burden on the health care system and the economy. Patients with osteoporotic fractures utilize more healthcare services, thereby imposing increased healthcare costs. Several health economic studies on the cost of osteoporosis have been published within the last two decades, especially in Europe and North America [6–8]. The majority of these are cost-of-illness studies (COI), which provide estimates cost of osteoporosis, but give no guidance on how to allocate resources to improve efficacy. The information given by COI on the societal burden of an illness is important for determining the awareness and attention the illness should be given [9, 10]. Studies on the cost of osteoporosis have primarily focused on hospital-related costs, thus no indirect costs are included. Modelling in health economics has several advantages because of the ability to extrapolate the results from studies, combining best evidence from multiple sources, and outline areas of uncertainty [11].

The aim of this study is to solely estimate the societal burden imposed by osteoporotic fractures in Denmark. In contrast to prior studies, this study will present a comprehensive model for the cost of osteoporotic fractures regarding hospitals, general practices, the municipalities, the regions and the patients.

## Materials and methods

This COI study applied an incidence-based bottom-up approach from a societal perspective, including both direct and productivity costs. This study took form of secondary analysis of relevant publications and administrative data from different Danish registers. To solely include osteoporosis-attributable costs, the incremental costs between osteoporotic patients and the corresponding background population were incorporated in the estimations of cost of osteoporotic fractures in Denmark. The term direct costs covers the costs of inpatient, outpatient, rehabilitation, nursing home, respite care, social services and assistive devices. The costs of productivity loss resulting from not being able to perform work, early retirement or death were calculated by the human capital approach. All costs included in the study are given in 2011 prices, and a gross costing approach has primarily been undertaken.

### Systematic literature search

A systematic literature search was performed, in accordance with PRISMA statement, to identify existing literature on COI regarding osteoporotic fractures. The search was conducted on September 25, 2012 in Cochrane Library, Embase, CINAHL, PubMed, EconLit, SveMed+ and Bibliotek.dk. Search terms, MeSH if possible, which were relevant to osteoporosis, fractures, costs and cost of illness, were applied, and equivalent Danish terms were used to search Bibliotek.dk. Inclusion criteria for this review were: men and/or women above 40 years; suffering from primary or secondary osteoporosis; hip, vertebral and/or wrist fractures; and cost of illness. The search identified 1,529 studies, and of these, 24 were identified as relevant. Six additional studies were identified from references, giving a total of 30 studies.

### Model approach

As osteoporosis is a chronic disease with recurrence of fractures, a Markov state transition model was appropriate to estimate the total societal cost of osteoporotic fractures in a Danish population of 50–99 years of age. Most orthopaedic surgeons often only diagnose fracture and not the cause of fracture (e.g. osteoporosis), thus to identify precise estimations of incidence of osteoporosis, fracture occurrence was combined with age [1]. From Danish literature, it was estimated that 17.7 and 40.8 % of Danish men and women over the age of 50 years are at risk of osteoporosis [1]. Thus, in 2011, the cohort should consist of 149,466 men and 388,474 women, which is the cohort used for Markov simulation in this study.

**Fig. 1:**
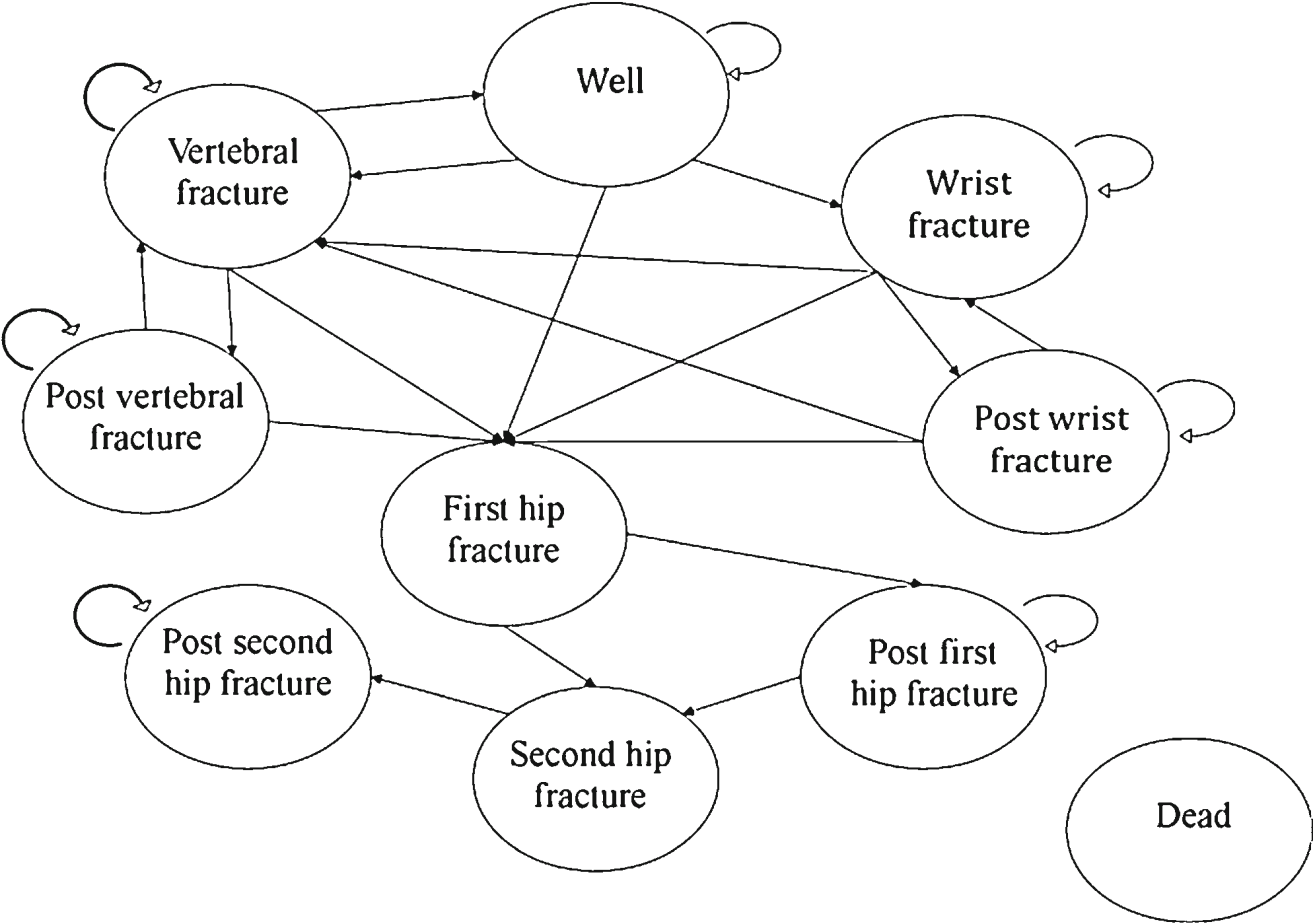
Illustration of the transition possibilities in the Markov model

A Markov model was constructed and analysed with TreeAgePro version 2012, TreeAge Software, Inc. The model simulated the natural history of osteoporotic fractures by tracking fracture events and costs for the cohorts of patients as they transition across the ten health states, using 1-year cycles. The health states included in this model were: well, wrist, post-wrist, vertebral, post-vertebral, first hip, post-first hip, second hip, post-second hip and death. This is illustrated in Fig. 1. The post-fracture states were included to minimise the “memory-less” feature of Markov modelling, hence the increased risk of fractures following first fracture and associated cost were incorporated in the model simulating the natural history of osteoporotic fractures. All patients in the cohort were assigned to only one state for each cycle. The model was limited to maximum two hip fractures during a lifetime. One model for each gender was constructed to capture the different cost and probabilities across genders. Tables were applied for transition probabilities as these may change over time.

All transition probabilities are based on data from The Danish National Hospital Discharge Register (DNHDR) combined with relative risks for new fractures [12] and death [13] and were transformed to gender- and age-dependent probabilities.

The Markov model will be used to estimate incremental lifetime cost of fractures discounted back to 2011 for both genders and this will be divided into cost per health state and into payer perspective. The calculations for the total cost of osteoporosis will be based on incremental lifetime cost and the demographic composition.

### Identification of model population

The selected model population was all Danish citizens ≥50 years with an osteoporotic fracture between 2001 and 2010, as osteoporotic fractures most often occur in this age group [6, 14, 15]. The model population was divided into different age groups at 5-year intervals, ranging 50–99. An osteoporotic fracture was defined as wrist fracture (ICD-10: S52, S52.5B and S52.9), vertebral fractures (ICD-10: S22.0-S22.1, S32.0-S32.2 and S32.7-S32.8) or hip fractures (ICD-10: S72, S72.0 and S72.1), as these were considered directly associated with osteoporosis [2, 3]. For vertebral fractures however, the true prevalence is expected to be underestimated, since the majority are morphometric [16]. A Danish study has shown that only 10 % of vertebral fractures were treated in a hospital, 40 % received pain relief at own general practitioner (GP) and 50 % were not diagnosed [17]. Thus, the numbers identified by diagnosis codes only represent the minority of vertebral fractures, whereas it must be assumed that all hip fractures and wrist fractures were diagnosed and treated [18, 19]. In the Markov model, all vertebral fractures, both those with and without clinical manifestations, are included. Second fractures of the wrist and vertebral are not included in this model, but are considered as a new fractures, as patient specific data were not available. Furthermore, readmissions were not possible to exclude as patient-specific data were not available.

### Data sources

In Denmark, all citizens are covered by the national public health system and all contact to hospitals is free and registered according to their social security number. All contacts, both in- and outpatient, are registered in DNHDR, from which it is possible to locate the incidence of different diseases, e.g. fractures [19]. Studies investigating the validity of DNHDR found that the validity of fracture reports was 97 % [18]. From the DNHDR, data on diagnosis and operation codes were drawn for 2001–2010 to estimate the incidence of fractures, corresponding treatment and rehabilitation [20]. In Denmark, 25 % of wrist fractures were treated with surgery [17, 21], and 25 % were referred to physiotherapist [17, 22]. As many vertebral fractures are undiagnosed, probably only 10 % were registered in DNHDR [17], hence the remaining fractures and resource use were estimated based on literature and clinical experts. In case of hip fracture, 75.4 % of women and 82.3 % of men were surgically treated using internal fixation methods [4], and the remaining fractures were treated with alloplastic. Two kinds of rehabilitation were offered in Denmark with 96.3 % having standard rehabilitation [20]. On average, hip fracture patients were treated at rehabilitation institutes 2 h/week for 3 months [23].

Furthermore, the length of stay (LOS) for the different fractures was collected from DNHDR and used to estimate the productivity loss for the working age group (50–64 years) (LOS: wrist, 1 day; vertebral, 4 days) [20]. Productivity loss following hip fracture was set to 3 months, as patients were not able to resume their previous activities and work immediately after discharge from hospital [24]. The relative risk for new fractures differed between genders and was estimated based on the literature [12, 25].

In order to calculate age-specific incidence rates for fractures and death, the census database from Statistics Denmark was used to retrieve population numbers and deaths from 2001 to 2010 [26]. Data from Statistics Denmark were also used for data collection in order to calculate productivity costs (see Table 1). The risk of dying following a fracture was retrieved from literature [13, 27, 28].

**Table 1:** All cost used to estimate cost of fractures, both direct and productivity costs

Cost component	Item	Unit Costs (EUR)	Data source
Direct cost			
Inpatient care			
Wrist	Orthopaedic care	2,189.01	[36]
	Emergency room care	224.40	
Vertebral	PVP	7,507.24	[36]
	Medical treatment	3,176.01	
Hip	Internal fixation	9,945.17	[36]
	Pertrochanteric internal fixation	6,327.75	
	Intracapsular alloplastic surgery	9,216.89	
DXA scan		350.27	[36]
GP^a^	Annual consultation	175.82	[37]
Pharmaceuticals^a^	Men (annual cost)	332.53	[38]
	Women (annual cost)	303.83	
Rehabilitation^a^	Standard care (hourly cost)	113.84	[23]
	At own home	0.00	
Respite care^a^	At nursing home (daily cost)	112.09	[44]
	Co-payment for patients	15.82	
Nursing home^a^	Yearly cost	51,858.98	[44]
Social services			
Food service	Cost per delivery, municipal	1.60	[40]
	Cost per delivery, co-payment	6.30	
Personal care	Cost per hour	62.26	[40]
Assistive devices			
Arthrodesis cushion	Purchase price	57.54	[45]
Bath bench	Purchase price	107.24	[45]
Bed raiser	Purchase price	19.30	[45]
Crutch	Purchase price	19.71	[45]
Dressing stick	Purchase price	40.48	[45]
Reaching aid	Purchase price	13.40	[45]
Toilet raiser	Purchase price	58.98	[45]
Walker	Purchase price	246.51	[45]
Productivity cost			
Productivity loss^a^ (daily wage per age group)			
50–54	Men	219.96	[26]
	Women	168.04	
55–59	Men	211.51	[26]
	Women	159.53	
60–64	Men	186.60	[26]
	Women	133.16	
65	Men	145.15	[26]
	Women	102.08	

No cost difference between men and women unless otherwise stated

^a^No cost difference between the types of fracture

Prescribed pharmaceuticals in Denmark are registered by the Danish Medicines Agency. The data cover 100 % of all prescriptions [29]. In Denmark, the GP must apply for reimbursement for each patients treated with anti-osteoporotic pharmaceuticals, except for alendronate in the case where a patient has had a low energy hip fracture [30]. However, these guidelines were only introduced recently and may thus have had little effect. It was presumed the persistence with the initial anti-osteoporotic pharmaceutical treatment was as stated in Roerholt et al. [31]. Calcium and vitamin D are neither prescribed nor reimbursed and purchases are thus not registered, so it was assumed that all patients using prescription pharmaceuticals are taking calcium and vitamin D supplement.

The Danish Longitudinal Study of Ageing consists of data from a national questionnaire survey performed in 1997, 2002 and 2007 on a sample of elderly Danish citizens aged ≥50 representative of their age groups. The database features a total of 23,704 completed questionnaires from 13,075 individuals. The three main areas of interest in this database are everyday life, labour market conditions and use of government benefits [32]. From this database, information regarding incremental use of personal care (16.6 %) and visits to GP (15.7 %) between osteoporotic and non-osteoporotic patients were retrieved. Hours per week of receiving personal care were estimated based on a Danish governmental report at 3.7 h [33].

Data regarding the use of assistive devices (walker, reaching aid, toilet raiser, dressing stick, crutch, bed raiser, bath bench and arthrodesis cushion) were retrieved from literature [34] and validated by experts. Further, 12 % of hip fracture patients received food service [34].

The following unpublished data from Odense University Hospital were kindly provided by M.D. Jesper Ryg: the incremental discharge to nursing home (i.e. compared to average Danish citizens) or discharge to respite care following hip fracture were 5 % for both. All future costs applied in the Markov model are discounted at 3 % annually as recommended by the National Institute for Health and Clinical Excellence [35].

### Estimation of costs associated with inpatient care

Hospital costs are accumulated in secondary sector and do not differ between genders or age groups. Inpatient costs for the three fracture types were determined from Danish diagnosis-related group (DRG) browser [36], which contain information on total reported DRG cases with corresponding ICD-10 diagnoses, cost weights and average LOS [36]. For vertebral fractures, which are not diagnosed, no cost will be applied. This is illustrated in Table 1.

### Estimation of costs associated with GP and pharmaceuticals

GP costs were accumulated in general practice and do not differ between gender or age groups. These included consultation fee, annual check-up, blood tests and telephone consultation [37].

Pharmaceuticals included prices for anti-osteoporotic pharmaceuticals, pain-relieving medication, calcium and vitamin D. From a calculated weight average purchase price for anti-osteoporotic pharmaceuticals, a patient would be reimbursed 75 % of purchase price [38]. This is illustrated in Table 1.

### Estimation of costs associated with rehabilitation, respite care and nursing home

Rehabilitation, depending on type of fractures and the patient’s health state, during admission was included in the DRG. After admission, rehabilitation takes place at specialized units as standard rehabilitation or rehabilitation in own home. Respite care is temporary care, on average 26.8 days after hip fracture (unpublished data, M.D. Jesper Ryg), at a nursing home facility for patients needing care after hospitalisation. As many elderly eventually move to a nursing home, only the incremental length of stay due to osteoporotic hip fractures was included in this study. The incremental length of stay at nursing home was estimated at 2 years, as the average age for moving to nursing home in general population was 84 years [39] and for osteoporotic patients it was 82 years (unpublished data, M.D. Jesper Ryg). Patient co-payment for nursing home was fixed at 10–20 % of the total costs of stay at nursing home depending on the personal income. The patient’s co-payment to food at the nursing home and cost for staying at respite care was also included. This is illustrated in Table 1.

### Estimation of costs associated with social services and assistive devices

Social services and assistive devices were only included for hip fracture patients because it was estimated that wrist or vertebral fracture itself does not increase the need for social services or assistive devices compared to the general population. Cost data of social services was retrieved from the National Board of Social Affairs including costs of food delivery and personal care [40]. Personal care is a social service offered in Denmark and includes help for personal hygiene, getting dressed, etc. Eight types of assistive device were included in the study and costs for assistive devices were estimated from the municipal suppliers. This is illustrated in Table 1.

### Estimation of productivity costs

As a societal perspective was adopted, the cost of absence at work following osteoporotic fractures was included. The human capital method was used to estimate the productivity loss by valuing the time away from the labour market as the average daily wage in Denmark, according to gender and age groups [26]. To estimate the productivity losses, the number of days absent from work due to hospitalisation, rehabilitation, respite care and nursing home or premature death (before retirement age at 65 years) was included. Valuation of an average daily income and the labour force participation rate for men and women between 50 and 65 years were estimated from Statistics Denmark [26]. All costs are illustrated in Table 1. Cost of informal care was not included.

### Validation of the Markov model

To assess the assumptions made for the COI model, several analyses were performed. Firstly, in order to confirm that the estimated number of fractures were the equivalent to those modelled by the programme, debugging was conducted. This debug method was done by applying tracker variables for the health states first hip, second hip, vertebral and wrist and comparing this result with the observed incidence from DNHDR. Secondly, to estimate which costs and probabilities would have the most impact on the COI, a one-way sensitivity analysis was conducted. For this analysis, intervals of likely best/worst scenarios were applied.

## Results

**Fig. 2:**
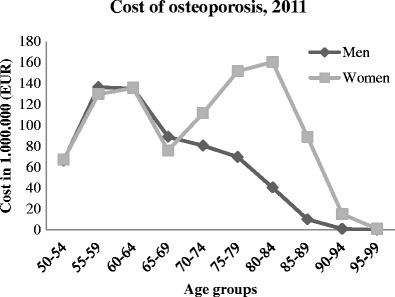
Total cost of osteoporotic fractures in 2011 divided into age groups and genders

The total cost of osteoporotic fractures in Denmark was estimated to EUR 1.563 billion in 2011, at EUR 628 million and EUR 936 million for men and women, respectively. This number was based on an estimated 149,466 men and 388,474 women at risk of osteoporosis. The total costs for different age groups are illustrated in Fig. 2. It should be noted that the peak for both men and women between 50 and 69 years could mainly be explained by productivity costs.

The incremental lifetime costs imposed by osteoporotic fractures, hence osteoporosis, were EUR 35,714 and EUR 26,152 per person for men and women ≥50 years in 2011, respectively. Furthermore, the age of 78 years for men and 84 years for women was the most costly.

### Fracture cost

When dividing the incremental lifetime costs into the health states to investigate the cost of different fracture types, following results emerged: First hip fracture was the most expensive at EUR 13,683 for men and EUR 9,385 for women, equivalent to 38 and 36 %, respectively, of the incremental lifetime cost accumulated for osteoporotic patients. Furthermore, well, wrist, post-wrist and post-vertebral may be considered relatively inexpensive health states compared to vertebral, post-first hip, second hip and post-second hip. All results on fracture costs are illustrated in Table 2.

**Table 2:** Incremental life time cost per person divided by health states

Health state	Men, EUR (%)	Women, EUR (%)
Well	42.44 (0.12)	128.85 (0.49)
Wrist	23.62 (0.07)	79.16 (0.30)
Post-wrist	3.32 (0.01)	114.91 (0.44)
Vertebral	2,129.51 (5.96)	2,462.37 (9.42)
Post-vertebral	41.77 (0.12)	479.11 (1.83)
First hip	13,682.59 (38.30)	9,385.04 (35.89)
Post-first hip	9,187.82 (25.73)	5,294.16 (20.24)
Second hip	5,759.17 (16.13)	4,678.48 (17.89)
Post-second hip	4,844.14 (13.56)	3,530.10 (13.50)
Dead	0.00 (0.00)	0.00 (0.00)
Total	35,714.38 (100.00)	26,152.18 (100.00)

### Who carries the burden?

The incremental lifetime costs of osteoporotic fractures were divided according to the payer, giving five categories, regional, general practice, municipalities, patient and productivity cost, which were carried by the employers and employees (the osteoporotic patients with fractures). The results are illustrated in Table 3. The municipal held the majority of incremental lifetime costs associated with osteoporotic fractures 55–57 %. Regional costs were associated with 22 and 25 % of incremental lifetime cost for men and women, respectively. Patient cost amounts to approximately 7 % for both genders. General practice cost amount to 0.67 % for men and 0.74 % for women. Productivity costs amount to 14 % for men and 13 % for women.

**Table 3:** Cost divided by payer

Payer	Elements	Men, EUR (%)	Women, EUR (%)
Regional	Medical treatment at hospital, 25 % of assistive devices and 75 % of pharmaceuticals	7,621.19 (21.34)	6,473.71 (24.75)
General practice	Treatment at GP	237.69 (0.67)	192.03 (0.74)
Municipal	Food service, personal care, 75 % of assistive devices, nursing home, respite care and rehabilitation	20,381.25 (57.06)	14,463.27 (55.31)
Patient	25 % of pharmaceuticals (though 100 % for pain relief of vertebral fractures), co-payment for nursing home and respite care and food service when living at nursing home	2,484.78 (6.96)	1,648.53 (6.30)
Productivity loss	Sick days caused by fracture and due to death	4,989.47 (13.97)	3,374.64 (12.90)
Total cost		35,714.38 (100)	26,152.18 (100)

### Validation of model

The one-way sensitivity analysis showed that only few variables had significant effect on the total incremental lifetime cost. The following results were true for both genders. From the analysis, it was observed that the amount of hours of personal care delivered, when these were varied from 3.4 to 6.55 h per week, changed the result to the highest level. The second most influential variable in the analysis was the cost for personal care. For men, the interval for change in incremental lifetime costs was between approximately EUR 31,000 and EUR 46,000, and for women, the interval was between approximately EUR 22,500 and EUR 33,000.

When performing the debug method on the model, roughly the same number of fractures was produced as expected from literature for both men and women. Because the model favours hip fractures over wrist and vertebral fractures, the two latter show lower fractures from the model than expected from DNHDR, while hip fractures are a bit more frequent than expected from DNHDR.

## Discussion

The societal burden imposed by osteoporotic fractures in Denmark in 2011 was estimated at EUR 1.563 billion. This result was divided according to payers, from which the Danish municipalities held the majority of the costs (55–57 % of total cost). Danish regions were the second greatest payer for osteoporotic fractures in Denmark at 21–25 % of total cost. When looking at the cost depending on the type of fracture, the most expensive health states were first hip and post-first hip accounting for 20–38 % of the total costs. The incremental lifetime cost imposed by osteoporotic fractures for men and women ≥50 year in 2011 was 35,714.38 EUR and 26,152.18 EUR per person, respectively.

Results showed that a man on average was more expensive than a woman. However, women consumed the highest total cost. This is because more women than men are at risk of developing osteoporosis (40.8 vs. 17.7 %) [1] and thus consume a greater total cost. When investigating the different costs across age groups, the most expensive age group was 75–79 for men and 80–84 years for women. This is a result of a larger fraction of patients in the expensive fracture or post-fracture states, compared to earlier age groups. Furthermore, in the stages after 75 years, the mortality increases for both gender, why the cost declines as a consequences hereof.

Similar studies have also investigated the cost of fractures. When investigating, e.g. hip fractures, other studies have shown results within the range of EUR 15,500–50,000 when converted to 2011 prices by consumer price index (for European countries, harmonized indices of consumer prices in health care have been used) [6, 14, 15, 41, 42]. This study showed that the estimated cost of hip fractures was EUR 13,683 for men and EUR 9,385 for women, which also included productivity cost. Thus, the result from this study must be considered very conservatively estimated and in the lower end of the interval compared to other COI studies.

In Denmark, the taxes finance the majority of the health care system, which includes treatment at hospital, GP, rehabilitation, etc. Because health care systems across the world are organised very differently (except for Scandinavia, UK, Germany etc. which are somewhat comparable to Denmark), it may be difficult to compare this study’s results of total cost from a payer perspective with other studies. However, hidden costs of osteoporotic fractures must be expected in countries with different models for the health care system economy, as well as those with similar organisation as the Danish.

The results of this study can help bring awareness to this major economic health problem that osteoporotic fractures constitute. This study is the first of its kind to cover the national economic burden in Denmark, and it is expected that the result will increase the attention to the disease, as several payers are substantially economically involved. The cost of osteoporotic fractures may earlier has been hidden as the health care costs of old age are significantly increased, and by covering the true incremental cost of osteoporotic fractures, osteoporosis should receive an increased societal awareness.

When decisions regarding screening or preventive treatments are made by decision makers, both costs and potential health gains must be considered. The complete societal burden of osteoporotic fractures should therefore also include quality of life, as this is an important aspect of patients' well-being. COI studies from Sweden [6, 9], New Zealand [41] and Germany [43] show that osteoporosis and osteoporotic fractures impose a significant loss in quality of life. Thus, it must be expected that similar findings would be found for the Danish population.

The strengths of this study include the empirical data used to estimate the number of fractures. Danish registers have a documented validity for diagnoses, especially fractures. Gender- and age-specific values have been applied within the model whenever possible, to include variation that may exist. Sensitivity analyses have been used to assess which assumptions would influence the cost of osteoporotic fractures and to produce validity. This COI study has applied a broad societal perspective, by including costs for social services, rehabilitation, nursing home, productivity loss both due to illness and death, etc. Thus, it must be deduced as a valid COI study. This should be compared to other COI studies of osteoporosis that most often only consider hospital perspective and/or do not include productivity loss.

Conservative estimations in this study may underestimate the actual societal cost of osteoporotic fractures. First of all, the study population only included patients above 50 years of age. However, a few may develop the disease or experience fractures before this age. Furthermore, only osteoporotic fractures have been used to estimate the societal cost, but these patients may have unknown expensive co-morbidities. Secondly, the types of fractures included can with certainty be ascribed osteoporosis. However, fractures may occur elsewhere but are not included in this study. This provides an underestimation of the total cost of osteoporosis, estimated by other COI studies to constitute approximately 3–19 % [14, 44]. Furthermore, it cannot be precluded that some of those fractures included are caused by metastases and should therefore have been eliminated. Thirdly, the quality of life has not been estimated or valued for this study population and cost for family caregiver time was not valued. Other sources than peer-reviewed journals have been used due to lack of cost data from these. Therefore, other sources such as Danish scientific articles and consultant reports have been used when necessary.

This study prove that the increased societal burden of osteoporotic fractures is substantial at EUR 1.563 billion and must be acknowledged nationally as an important health problem. Medical costs of the osteoporotic fractures are substantial cost for the health care sector, but are by far exceeded by the cost for the municipal in terms of social services and rehabilitation. In fact, the 55 % of the burden of osteoporotic fractures is carried by the municipalities. The broad impact of the disease across age and gender underlines the need for treatment and awareness to the population with growing and unrecognized needs.

## Electronic supplementary material

Below is the link to the electronic supplementary material.

ESM 1 (DOCX 28 kb)
