# Supplementary material for: A health economic analysis of osteoporotic fractures: who carries the burden?
Source: Arch Osteoporos. 2013 Feb 19;8(1):126. doi: 10.1007/s11657-013-0126-3 (PMC3880482; doi:10.1007/s11657-013-0126-3)
Supplement: Supplementary file 1 — (DOCX 28 kb) [file 11657_2013_126_MOESM1_ESM.docx]

**A Health Economic Analysis of Osteoporotic Fractures;
who carries the burden?**

Louise Hansen, MSc^1^*, Anne Sofie Mathiesen, MSc^1^*, Peter Vestergaard, Professor MD PhD DrMedSc^2,3^, Karin Dam Petersen MPH PhD^1^, Lars H. Ehlers Professor PhD^1^

*These two authors contributed equally to the work.

^1^Danish Center for Health Care Improvement, Aalborg University, Aalborg, Denmark

^2^Faculty of Medicine and Health Science Technologies, Aalborg University, Aalborg, Denmark

^3^Department of Endocrinology, Aalborg University, Aalborg, Denmark

**ESM 1:** Description of variables used in the Markov model

| Type of variable | Specification | Variable value | Data source |
| --- | --- | --- | --- |
| Risk of osteoporosis | Men  Women | 0.177  0.408 | [1] |
| Treatment   - Wrist fracture* - Vertebral fracture - Hip fracture | Orthopaedic care  PVP, men  PVP, women  Medical treatment, men  Medical, women  Internal fixation, men  Internal fixation, women | 0.25  0.0262  0.0226  0.0738  0.0774  0.8225  0.7542 | W[17], [21]  V[17], [19], [20]  H[4] |
| Discharge to nursing home* | Following hip fracture | 0.05 | Unpublished data, Jesper Ryg |
| Discharge to respite care* | Following hip fracture | 0.05 | Unpublished data, Jesper Ryg |
| Rehabilitation   - Wrist fracture* - Vertebral fracture* - Hip fracture* | Physiotherapist  Standard rehabilitation  Standard rehabilitation | 0.25  0.9629  0.9629 | W[17], [22]  V[20], [23]  H[20], [23] |
| Social services   - Personal care* - Personal care* - Visits to GP* - Food service* - Assistive devices* | Probability  Frequency, hours per week  Probability  Probability  Arthrodesis cushion  Bath bench  Bed raiser  Crutch  Dressing stick  Toilet raiser  Walker | 0.166  3.7  0.157  0.12  0.24  0.52  0.31  0.72  0.24  0.52  0.57 | [33], [34], [46] |
| Relative risk for new fracture  Men:   - Wrist fracture - Vertebral fracture - Vertebral fracture - Hip fracture - Hip fracture - Hip fracture   Women:   - Wrist fracture - Vertebral fracture - Vertebral fracture - Hip fracture - Hip fracture - Hip fracture | Following previous wrist  Following previous wrist  Following previous vertebral  Following previous wrist  Following previous vertebral  Following previous hip  Following previous wrist  Following previous wrist  Following previous vertebral  Following previous wrist  Following previous vertebral  Following previous hip | 3.6  10.7  33  2.7  4.7  2.2  3.3  1.7  4.4  1.9  2.3  2.2 | [12], [25],[47], [48] |
| Relative risk of dying following fracture for age groups 50-54 and 55-59**  Men:   - Wrist fracture - Post wrist fracture - Vertebral fracture - Post vertebral fracture - Hip fracture - Post hip fracture   Women:   - Wrist fracture - Post wrist fracture - Vertebral fracture - Post vertebral fracture - Hip fracture - Post hip fracture |  | 0.7  0.9  4.4  1.35  3.4  3.65  0.4  0.9  3.4  1.85  21.3  2.75 | [4], [13], [28] |
| Productivity loss   - Wrist fracture* - Vertebral fracture* - Hip fracture* | Frequency  Frequency  Frequency | 1 day  4 days  3 months | W[20]  V[20]  H[24] |

*No difference between genders.
**Relative risks of dying following fracture were applied according to age groups.

W=wrist, V=vertebral, H=hip
